# Miniaturized electrocoagulation approach for removal of polymeric pigments and selective analysis of non- and mono-hydroxylated phenolic acids in wine with HPLC-UV†

**DOI:** 10.1039/d0ra09089a

**Published:** 2021-02-02

**Authors:** Kanokporn Chindaphan, Isaya Thaveesangsakulthai, Suchapa Naranaruemol, Thumnoon Nhujak, Janjira Panchompoo, Orawon Chailapakul, Chadin Kulsing

**Affiliations:** Department of Chemistry, Faculty of Science, Chulalongkorn University Bangkok 10330 Thailand ckulsing@gmail.com; Chromatographic Separation and Flavor Chemistry Research Unit and Center of Molecular Sensory Science, Department of Chemistry, Faculty of Science, Chulalongkorn University Bangkok 10330 Thailand; Electrochemistry and Optical Spectroscopy Center of Excellence (EOSCE), Department of Chemistry, Faculty of Science, Chulalongkorn Universit Bangkok 10330 Thailand

## Abstract

Electrocoagulation (EC) approach was developed to allow fast sample cleanup step prior to selective analysis of non- and mono-hydroxylated phenolic acids in red wine samples with high performance liquid chromatography hyphenated with UV detection (HPLC-UV). EC system with the wine in KCl_(aq)_ electrolyte (1.5 mol L^−1^) was employed removing the polymeric pigments with good recovery of 39 peaks from 64 peaks. The mechanisms mainly involve enrichment induced aggregation and reduction of the pigments at the cathode and the adsorption onto the EC sludge. The EC was further miniaturized employing two intercalated stainless steel spring electrodes at 9.0 V which allowed removal of >99% interference peak area from the pigments within 5 s. The recoveries of the target phenolic acids (*p*-hydroxybenzoic acid, vanillic acid, syringic acid and ferulic acid) were within the range of 86–102%. The repeated analysis of these standards revealed <2 and ≤10% RSD of the intra-day and inter-day precisions, respectively. The linearities of their calibration curves were observed with *R*^2^ > 0.99. Their method detection limits were within the range of 0.02–0.20 mg L^−1^.

## Abstract 2

Electrocoagulation (EC) approach was developed to allow fast sample clean-up step prior to selective analysis of non- and mono-hydroxylated phenolic acids in red wine samples with high performance liquid chromatography hyphenated with UV detection (HPLC-UV).

## Graphical abstract

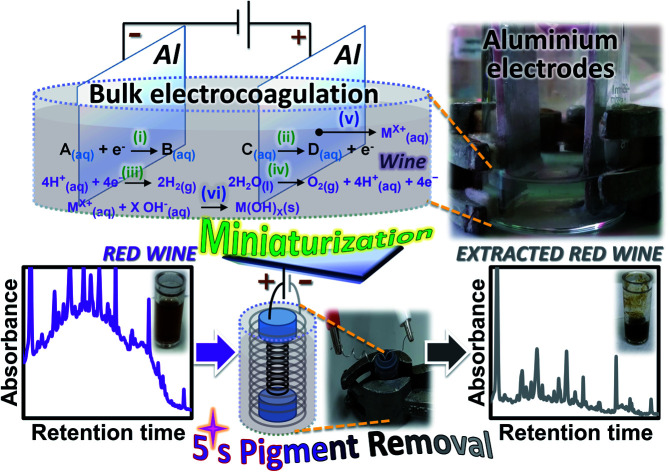

## Introduction

Wine samples are very complex containing several hundred volatile^1^ and non-volatile compounds.^2^ Among different species, non- and mono-hydroxylated phenolic acids such as cinnamic acid, *p*-hydroxybenzoic acid, vanillic acid, syringic acid, ferulic acid and sinapic acid are of interest due to their antioxidant, antimicrobial, anti-inflammatory, anticancer or antianxiety activities or prevention of bone degeneration.^3^ Analysis of these compounds in red wine using liquid chromatography, UV-visible spectroscopy or their hyphenation often suffers from the low polar interference, especially the pigments and the related species observed as an increasing baseline at the end of reverse phase HPLC analysis.^4,5^ These interference species form during wine maturation and aging and are mainly anthocyanin derived pigments, such as polymeric proanthocyanidins or pyranoanthocyanins produced from condensation between anthocyanin and/or flavan-3-ols with the presence of aldehydes in wine.^6^ Applications of liquid chromatography-tandem mass spectrometry (LC-MS/MS)^7,8^ or sample derivatization prior to gas chromatography-MS^9,10^ are thus required to analyze these phenolic acids. The interference can also be removed by sample fractionation, solid phase extraction, ultrafiltration or ultracentrifugation approaches prior to HPLC-UV analysis.^11,12^ Alternatively, molecularly imprinted polymers have been synthesized and applied for selective extraction of target phenolic compounds.^13^ Although the multiple template imprinting approach was also reported^14^ allowing selective extraction of several target compounds, the approach requires synthetic skills, consumption of washing/eluting solvents, several minutes of sample pretreatment step and not directly applicable for analysis of a broad range of the non- and mono-hydroxylated phenolic acids.

Electrolysis is a reliable and cost effective approach with the recycling and monitoring capabilities applied to synthesize a wide range of organic compounds.^15^ Electrochemical oxidation of phenolic compounds generated their oxidized forms with the polymeric species^16^ which can be irreversibly adsorbed onto the electrode surface.^17^ In addition, electrolysis of wine under strong alternative electric field (600 V cm^−1^) was reported to allow fast wine aging process without use of flor as observed with the increasing contents of amino acids.^18^

Electrocoagulation (EC) is a process to treat wastewater, *e.g.* by removals of organic wastes, metal ions and the others.^19^ EC system relies on either electrochemical conversion of chemical wastes into the solid forms at the electrodes or corrosion of the applied electrodes resulting in formation of the solid complexes (sludge), *e.g.* formed as a result of complexation between metal ions from the corroded electrode and hydroxides^20^ with the subsequent adsorption of the wastes onto the sludge and their removal. Since corrosion is required in EC, relatively low cost electrode materials such as aluminium or stainless steel can be applied. Due to the capability to remove impurity from a sample with the selectivity depending on applied voltage, a challenge is to apply EC as selective sample preparation approach in the area of food and beverage analysis especially for extraction of target components from the complex matrix of red wine.

In this study, simple and fast EC approaches were developed and applied for removal of red wine pigments and selective analysis of phenolic acids. Effects of EC time and voltage were investigated. Possible extraction mechanisms were proposed according to electrolysis, EC and differential pulse voltammetry (DPV) results. Suitable condition was selected and applied for wine extraction and the results were discussed according to different non-volatile compound profiles of the extracted samples analyzed by HPLC-UV. The approach was further developed as a fast clean-up step and designed into a small-scale platform, evaluated according to selectivity, recovery, linearity range, limit of detection and repeatability and applied for analysis of the target compounds in different wine samples.

## Results and discussion

**Fig. 1:**
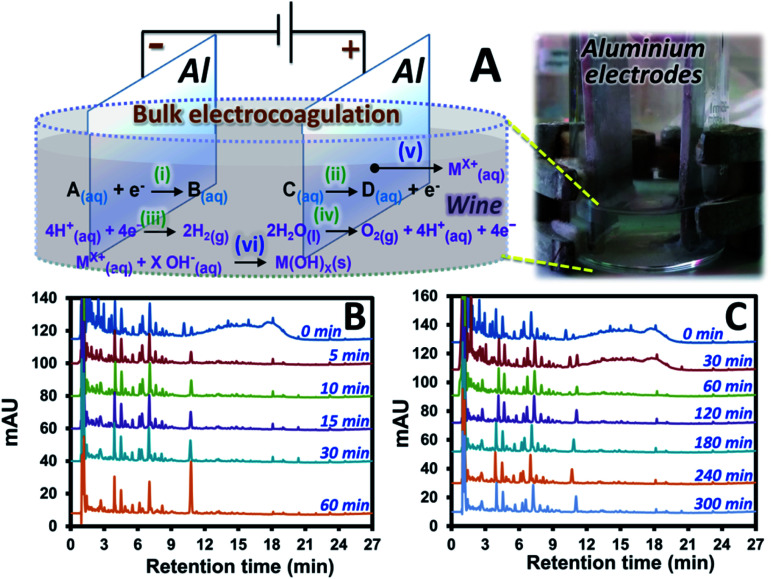
Overall bulk electrocoagulation studies: (A) experimental setup and chromatograms of wine A sample containing 1.5 mol L^−1^ of KCl_(aq)_ collected at different time of electrocoagulation at (B) 4.0 V and (C) 1.0 V. M = metal and *X* = absolute oxidation number of the metal.

Electrocoagulation (EC) based sample preparation approaches were established with the samples before and after the treatments analyzed by using HPLC-UV. Electrolysis (employing Pt electrodes) of a red wine sample (wine A) was initially investigated. This was compared with EC (using Al electrodes) of the same sample (Fig. 1B and S1C† with 30 and 60 min EC time). The suitable EC condition was then selected, and related sample clean-up mechanisms were proposed with the support from the EC and DPV experiments. Furthermore, a small scale electrocoagulation device was designed, validated and applied for clean-up of different wine samples and a mixture of standard compounds potentially observed in red wine. The extraction performance was discussed according to interference signal removal, recovery, precision and selectivity towards different target compounds.

### Electrolysis of standard mixture and wine

The system in Fig. S1A (see also the Experimental section S1†) was applied for electrolysis of wine A without addition of the electrolyte. The constant voltage mode was used for the sample treatment. This was initially investigated since the sample clean-up method development is expected to be as simple as possible with minimized reagent consumption. For the electrolysis of wine A (pH 3.0) on the stable Pt electrodes, oxidation and reduction of different species occurred, also see the mechanisms (i–iv) in Fig. S1A.† Since the dominant reaction was proton reduction as observed with the larger amount of H_2_ gas bubbles at the cathode compared with O_2(g)_ at the anode, the wine solution pH increased to 7.0 after 120 min of the electrolysis.

In order to generate significant current detectable with the power supply capability (0.003–0.005 A), a high voltage (9.0 V) was applied for different period of time. The samples before and after electrolysis were then analyzed with HPLC-UV with the results shown in Fig. S1.† Apart from O_2_ and H_2_ evolution,^19,21^ the longer electrolysis of wine A resulted in decreased peak areas of most of the species (Fig. S1B and C†) indicating their electrochemical conversion into the other species or adsorption onto the electrode as reported by the previous study, *e.g.* involving formation of polymeric species according to oxidation of phenolic compounds,^16^ as well as the formation of the brown solid species at the Pt cathode. In addition, the electrolysis revealed increasing areas of the peaks at lower retention time (<3 min) which indicated the polar oxidized species with the UV spectra with the absorbance below 240 nm including some amino acids previously reported for wine electrolysis using high electric field.^18^ To this end, the 9.0 V electrolysis removed most of the phenolic compounds and generated some polar oxidized species, and is not suitable for sample clean-up application.

With the aim to selectively remove the interference with the remaining target analytes, electrolysis at lower voltage is performed. NaCl (2.6 mol L^−1^) is thus added into wine A to enhance current at lower voltage. This allowed use of the lower voltage (4.0 V) with the initial current of 0.011 A. The HPLC results for the electrolysis of wine A at different time were shown in Fig. S1C.† However, the electrolysis still eliminated most of the compounds and generated some oxidized species with insufficient selectivity in the sample clean-up, albeit with the longer electrolysis time required for the similar results. In addition, the pigment interference observed as a high background signal (plateau) between the retention time of 9 and 21 min was not effectively removed. Possible compound classes responsible for the interference plateau in red wine have been previously reported as the polymeric pigments of anthocyanins observed as a hump at the end of reverse phase HPLC analysis such as that formed by reaction between flavan-3-ol (*e.g.* catechin/epicatechin) and anthocyanin glucosides (*e.g.* malvidin-3-glucoside).^11,22^ Gradient normal phase HPLC can be applied to separate this interference from the other species such as flavonols and oligomeric proanthocyanidins.^23^ However, the capability to separate hydrophobic compounds is low.

### Electrocoagulation of wine and the condition selection for selective sample clean-up process

The Pt electrodes were replaced with Al electrodes with larger size in order to perform electrocoagulation with the setup shown in Fig. 1A. This is performed in order to: (1) maintain the significantly high current at lower voltage, (2) reduce compound oxidation rate by increasing the oxidation pathway to electrode corrosion, see mechanism (v) in Fig. 1A, and (3) introduce formation of metal oxide (see mechanism (vi)) and sludge, with the subsequent adsorption of the pigments onto the sludge which would support sample clean-up process. Interestingly, under the same voltage of 4.0 V as that applied for the electrolysis, EC resulted in much higher initial current (1.894 A) and survival of several compounds in wine A after the EC as shown by the results in Fig. 1B. These remaining peaks of wine A after the EC showed the UV spectra similar to that of phenolic acids which are the key target compounds in this study. In addition, the pigment interference signal was mostly removed after 5 min EC. It should be noted that the longer EC time (60 min) resulted in the increasing peak areas of a few target compounds (*e.g.* see the peak of *p*-coumaric acid with the retention time of ∼11 min in Fig. 1B). Too long EC time is thus not suitable for sample clean-up application. Lower EC voltages were also investigated. 2.5 V EC at different time showed similar results (Fig. S2†) to that observed with the 4.0 V EC where the pigments were effectively removed within 5 min. The lower EC voltage (1.0 V) showed longer EC time to remove most of the pigments (see the chromatogram with the EC time of 60 min in Fig. 1C). However, the similar set of the compounds with similar peak areas was observed after the interference removal, compared to that at the higher voltage (see Fig. S2† or Fig. 1B with the 5 min EC). In addition, EC voltage effect was investigated at fixed EC time of 30 min. This revealed more interference removal as well as more H_2_ gas and sludge formation at the higher EC voltage with the resulting chromatograms as shown in Fig. S3.† Use of higher voltage also resulted in larger current and higher pH of the system.

From the results above, the interference and several compounds in wine A were selectively removed under significantly high voltage and long EC time offering selective sample clean-up for the target compounds that are more stable or inert under the applied EC condition. These compounds included several phenolic acids and flavanols which were confirmed by standard injection, comparison of the experimental UV spectra and the retention pattern with the literature data.^11,24–29^ In addition, the interference could be effectively removed by using EC with either “high voltage (high current) and short time” or “low voltage (low current) and long time” enabling significant amount of charge (*e.g.* current × time) for the sample clean-up.

### Proposed extraction mechanisms

**Fig. 2:**
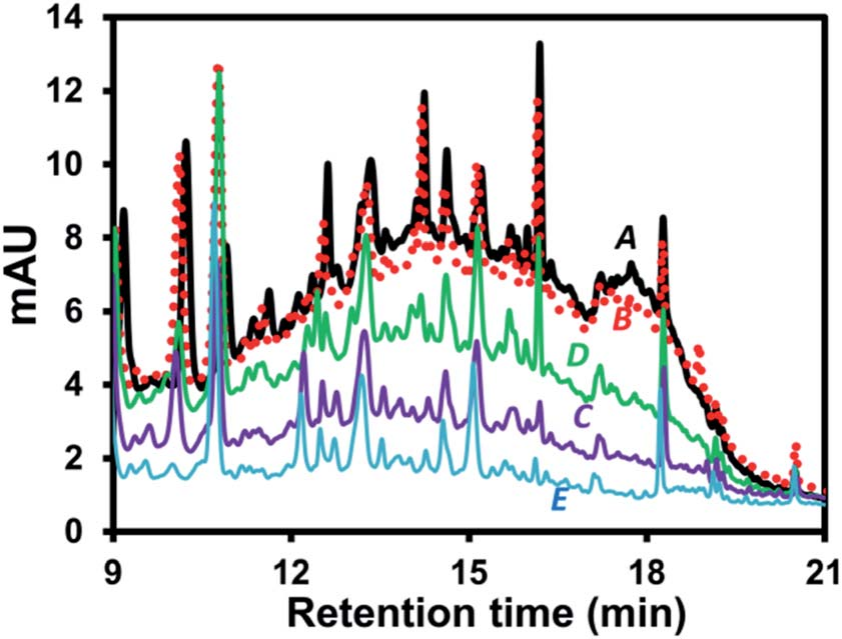
Chromatograms of: (A, black thick solid line) wine A and KCl_(aq)_ (1.5 mol L^−1^) at pH 3.0 without EC (the control sample) and the same samples (B, red dotted line) adjusted to pH 6.5 using NaOH without EC, (C, purple solid line) added with the sludge obtained from the other EC of KCl_(aq)_ without wine A, (D, green solid line) collected at the anode during bulk EC at 1.5 V for 20 min, and (E, blue solid line) collected at the cathode during bulk EC at 1.5 V for 20 min, illustrating electrocoagulation based sample clean-up mechanisms.

Possible mechanisms can be proposed to explain wine sample clean-up by the developed EC approach. The related mechanisms can be (1) water hydrolysis mainly resulting in H_2(g)_ at the cathode with smaller amount of O_2(g)_ at the anode, see mechanisms (iii) and (iv), respectively, in Fig. 1A. This resulted in the overall removal of H^+^_(aq)_ (increasing relative concentration of OH^−^_(aq)_) as confirmed by the increasing pH of the wine sample after the EC. It should be noted that the pigment interference could not be removed by simply adjusting the wine pH to the basic condition as shown by the similar area of the interference hump in HPLC-UV of wine A added with KCl and NaOH (Fig. 2B) compared with the control (Fig. 2A). Thus, the sample clean-up mechanism was not driven by basicity of the sample, see also the similar HPLC results observed at different pH in Fig. S4.† (2) The anode corrosion was observed producing metal ions such as Al^3+^_(aq)_ for the aluminium electrodes (mechanism (v) in Fig. 1A) or Zn^2+^_(aq)_, Fe^2+^_(aq)_, Fe^3+^_(aq)_ or Cr^3+^_(aq)_ for the stainless steel electrodes. Metal hydroxides can then be generated according to complexation between these metal ions and OH^−^_(aq)_,^17^ see mechanism (vi) in Fig. 1A, with (3) the subsequent formation of floating floc and sludge. These could induce either coagulation of the polymeric pigments in wine or their adsorption onto the sludge leading to the pigment removal from the solution. This purely induced coagulation/adsorption mechanism was confirmed by the significant decrease in the interference peak area (Fig. 2C) of the sample prepared by mixing the sludge obtained after EC of KCl_(aq)_ (without wine A) into wine A without the EC treatment. Furthermore, there can be (4) electrochemical oxidation and reduction of wine compounds resulting in different products, such as amino acids^18^ and the other oxidized compounds eluting before 3 min with the increasing peak areas after the EC treatment (*e.g.*Fig. 1) and polymeric species from some phenolic compound oxidation and polymerization^16^ followed by deposition onto the electrodes^17^ (leading to the decreasing areas of several peaks after the electrolysis treatment, *e.g.* Fig. S1B and C†). Thus, these products could not be the target analytes with the established EC clean-up approach. However, careful selection of the EC voltage and time offered selective removal of the polymeric pigments leading to the clean signals of target compounds in the HPLC result (*e.g.*Fig. 1B and C). It should be noted that the pigment removal mechanism was partly caused by their electrochemical oxidation as shown by the slightly smaller area of the interference hump in the chromatogram of wine A added with KCl collected during the EC at the anode (Fig. 2D compared with Fig. 2A). The polymeric pigments were positively charged which can be expected to undergo (5) enrichment and the subsequent aggregation at the (negatively charged) cathode as discussed in the previous studies investigating electric field induction effects in the environmental^30^ or clinical^31^ areas. The interference removal was mainly caused by either reduction or enrichment induced aggregation at the cathode as observed by the smallest area of the interference hump in the chromatogram of the sample collected during the EC at the cathode (Fig. 2E).

### Miniaturization of the EC system

**Fig. 3:**
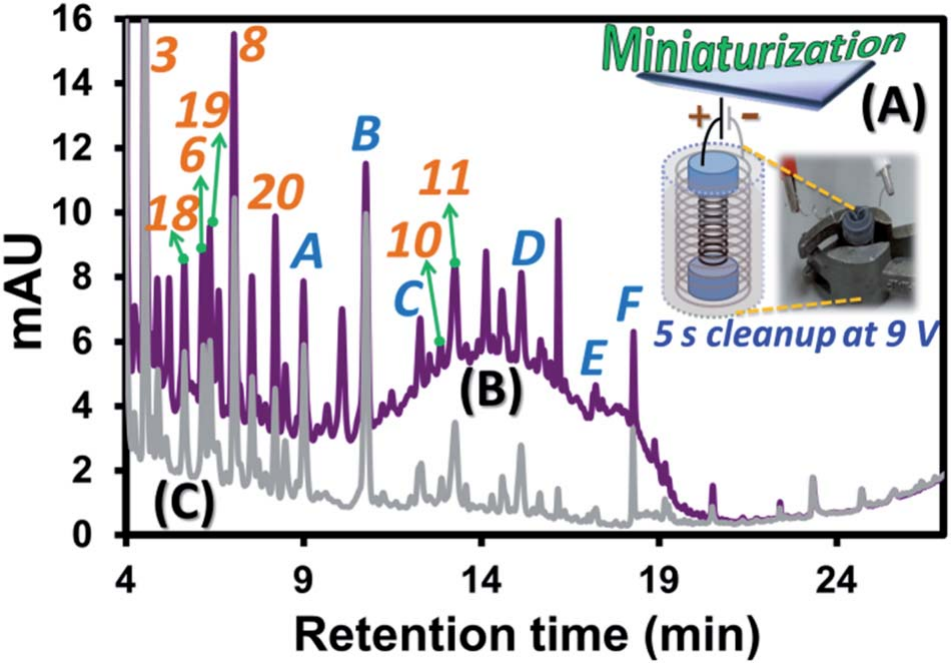
Diagram illustrating experimental approach: (A) small scale EC in a 2 mL vial, with the HPLC chromatograms at 270 nm of wine A sample containing 1.5 mol L^−1^ of KCl_(aq)_, (B) before and (C) after the EC clean-up. The labeled compound identities were provided in Table 3.

Since the developed EC approach requires solely a power supply and two corroding electrodes with wine and KCl_(aq)_ as the electrolyte, this can be redesigned into a smaller platform with more effective clean-up performance employing high contact surface area of the electrodes with wine sample. To this end, two stainless steel springs with different sizes where the smaller one can be inserted into the larger one (see black and grey springs, respectively, in Fig. 3A). Note that two ends of the smaller spring were wrapped with two pieces of parafilm before the insertion as shown by two blue cylindrical regions at the two ends of the black spring in Fig. 3A. These electrodes were well fit into dimension of a 2 mL vial. This system allows effective use of the vial space which critically increased the contact surface area per sample volume compared with the bulk EC (Fig. 1A). This is illustrated by much higher EC current per mL of wine solution (0.130 A mL^−1^) compared with that of the bulk EC (0.010 A mL^−1^) at 2.5 V. With aim to develop EC as a fast and effective sample clean-up process for the target compound analysis and interference removal, the “high voltage and short time” approach was applied with the selected EC voltage and time of 9.0 V and 5 s. Example use of the miniaturized system to reduce 99% peak area of the polymeric pigment interference from the wine A sample is illustrated by the two chromatograms before (Fig. 3B) and after (Fig. 3C) the clean-up. Note that the voltage was selected to be compatible with the conventional 9.0 V battery and the 5 s period was the selected time required to effectively remove (99%) the pigment interference, see the HPLC results with the relative areas of the interference observed by using different time of the miniaturized EC at 9.0 V in Fig. S5.† According to the proposed mechanisms above, the sample clean-up with this miniaturized system is mainly caused by enrichment induced aggregation and reduction of the pigments at the cathode and their adsorption onto the sludge. The reduction mechanism of the system consisting of two stainless steel spring electrodes connected to a potentiostat (Fig. S6A and the Experimental section S2†) was further investigated with DPV. Three repeated analyses of wine A with only the first scan recorded and overlaid as shown in Fig. S6B.† There was a reduction peak observed at *ca.* −0.7 V (*vs.* Ag/AgCl) during the DPV analysis of wine A, while no reduction feature could be observed for the blank solution. As this cathodic peak solely occurred with the presence of wine A in the electrolyte, it might be potentially related to reduction of oxidized phenolic compounds^32^ as well as reduction of anthocyanins and pyranoanthocyanins^33–35^ leading to removal of the interference peak of these species from wine A prior to the HPLC analysis. In addition, the result indicates the minimum required voltage of −0.7 V (*vs.* Ag/AgCl) to be applied for effective pigment removal. Note that the cathodic peak current obtained was not strictly reproducible for subsequent measurements, possibly due to the removal of the electrode coating (such as Zn_(s)_) after the 1^st^ experiment, revealing more active surface area of the electrode in the 2^nd^ and 3^rd^ experiments (as the same stainless steel spring electrodes were used for repeating the experiments in triplicate), and the slightly different amounts of the active substances in wine in each measurement.

### Evaluation and application of the developed device

The miniaturized device (Fig. 3A) using the 9.0 V and 5 s EC approach was evaluated with the application for clean-up of the standard mixture and wine A in KCl_(aq)_ prior to the HPLC analysis. The application was also repeated using three different EC devices each of which was repeated three times within the same day resulting in *n* = 9 in total. The evaluation results for the standard compounds and the interference in wine A were provided in Table 1. The very low recovery of the polymeric pigment (<1%) indicates effective sample clean-up process within 5 s. Most of the investigated standard compounds were removed together with the pigment interference after the EC. However, six compounds were survived with high recoveries (77–102%, see compounds 3, 5, 6, 8, 10 and 11 in Table 1) revealing selective clean-up result obtained from the developed EC approach. These target compounds also showed good intra-day and inter-day precisions (<2% and ≤10%, respectively) except for sinapic acid (11) with the higher % RSD values of 6 and 18%, respectively. This correlates with the lower recovery (77%) of this compound compared with the other target compounds. Note that the 6 compounds shown in Table 1 were just the example showing good recoveries based on the available standards. Good recoveries were actually observed for more related peaks in the wine sample (39 out of 64 peaks in Fig. S7†) indicating selectivity towards the phenolic acids. In addition, the good linearity of the calibration curves (0.5–100 mg L^−1^, with *R*^2^ of 0.9905–0.9981 for the 6 compounds showing good recoveries) and low method detection limit (Table 1) were observed.

**Table 1:** Evaluation data for application of the miniaturized EC system at 9 V for 5 s (*n* = 9) for treatment of 20.00 mg L^−1^ of the standard mixture with the polymeric pigment interference recovery obtained from wine A treatment analysis. The target compounds were emphasized with the bold and italic fonts

No.	Compound	*t*_R_ (min)	Extracted concentration (mg L^−1^)	Recovery from the EC clean-up^a^ (%)	Intra-day precision (% RSD)	Inter-day precision (% RSD)	Method detection limit^c^ (mg L^−1^)
1	Gallic acid	1.71	1.64 ± 1.00	7.91 ± 4.81	1.94	64.54	—
2	Protocatechuic acid	2.98	0.64 ± 0.14	3.10 ± 0.70	1.15	21.75	—
***3***	***p-Hydroxybenzoic acid***	***4.89***	***17.54 ± 1.63***	***86.11 ± 8.84***	***1.34***	***9.30***	***0.10***
4	Chlorogenic acid	5.28	0.25 ± 0.07	1.20 ± 0.31	5.30	27.58	—
***5***	***Caffeine***	***5.73***	***19.80 ± 0.72***	***96.86 ± 4.16***	***0.17***	***3.66***	***0.02***
***6***	***Vanillic acid***	***6.19***	***20.29 ± 2.10***	***99.35 ± 9.76***	***1.74***	***10.35***	***0.02***
7	Caffeic acid	6.67	0	0	0	0	—
***8***	***Syringic acid***	***7.09***	***20.79 ± 0.44***	***101.99 ± 3.38***	***0.77***	***2.12***	***0.05***
9	Ethyl gallate	10.14	0.97 ± 0.26	4.77 ± 1.18	2.07	26.26	—
***10***	***Ferulic acid***	***12.67***	***19.49 ± 0.49***	***95.56 ± 3.47***	***0.64***	***2.49***	***0.10***
***11***	***Sinapic acid***	***13.15***	***15.54 ± 2.85***	***76.58 ± 14.80***	***5.73***	***18.34***	***0.20***
12	Rutin	14.02	0.76 ± 0.33	3.71 ± 1.58	1.91	43.58	—
13	Ethyl protocatechuate	15.77	1.96 ± 2.28	9.52 ± 11.11	58.63	116.58	—
14	Myricetin	16.76	1.04 ± 0.03	5.31 ± 0.32	0.28	2.66	—
15	Resveratrol	17.70	0.94 ± 0.50	4.65 ± 2.44	6.05	52.66	—
16	Quercetin	18.86	0.53 ± 0.14	2.63 ± 0.70	3.66	26.37	—
17	Kaempferol	20.05	1.93 ± 0.06	9.66 ± 0.43	0.13	3.35	—
	Polymeric pigment	9–21	—	0.50 ± 0.41^b^	8.71	10.59	—

a Recovery evaluated by means of average concentration ratios of a compound in the samples after and before the EC clean-up.

b Recovery calculation based on average total peak area of the interferences.

c Method detection limit calculated according to dilution approach until the analyte peak height was approximately 3 times of the noise level. *t*_R_ = retention time, RSD = relative standard deviation.

Although there were several mechanisms for the polymeric pigment removal proposed in the above section, recoveries of the smaller phenolic compounds showed general correlation with their oxidation resistance. Phenolic compounds are antioxidants which can be oxidized into the other species decreasing their recovery from the EC clean-up. Thus, more electrochemically stable compounds with higher oxidation potential are expected to show higher recoveries. Caffeine (5) was also investigated as a less active compound towards oxidation with 97% recovery from the EC. A general functional group of the investigated phenolic compounds governing their oxidation behaviors is the hydroxyl group at the *p*-position relative to different functionalities, *e.g.* –OH of small phenolic compound, –COOH and –CH=CHCOOH of phenolic acids, the ring A (or ring B) of stilbenes with the *p*-hydroxyl group at the 4′ position on the ring B (or the 4 position on the ring A), or the ring C of the flavonoids with the *p*-hydroxyl group at the 4′ position on the ring B. This *p*-hydroxyl group can be more reactive with the presence of electron donating group in the effective (*o*- and *p*-) positions and less reactive with the electron withdrawing group.^36^ Electrochemical stabilities of the compounds here can be related to their structures relative to the *p*-hydroxyl group as shown in Table 2. Without additional electron donating groups, *p*-hydroxybenzoic acid (3) survived with the high recovery of 86%. With this finding, other compounds without or with a single hydroxyl group such as benzoic acid, cinnamic acid and *p*-coumaric acid are also expected to result in high recoveries from the EC clean-up.

**Table 2:** Different functional groups and their positions relative to the *p*-hydroxyl group in different analytes with the recoveries for comparison. The target compounds were emphasized with the bold and italic fonts

No.	Compound	ē donating group		*p*-Functional group	Conju-gation^b^	Steric effect^c^	Recovery (%)
		Effective^a^	Ineffective				
1	Gallic acid	2× (–OH)	—	–COOH	—	—	7.9
2	Protocatechuic acid	–OH	—	–COOH	—	—	3.1
***3***	***p-hydroxybenzoic acid***	***—***	***—***	***–COOH***	***—***	***—***	***86.1***
4	Chlorogenic acid	–OH	—	–CH=CHCOOR	—	—	1.2
***5***	***Caffeine*** d	***—***	***—***	***—***	***—***	***—***	***96.9***
***6***	***Vanillic acid***	***–OCH***_***3***_	***—***	***–COOH***	***—***	***✓***	***99.4***
7	Caffeic acid	–OH	—	–CH=CHCOOH	✓	—	0
***8***	***Syringic acid***	***2× (–OCH***_***3***_ ***)***	***—***	***–COOH***	***—***	***✓***	***102.0***
9	Ethyl gallate	2× (–OH)	—	–COOCH_2_CH_3_		—	4.8
***10***	***Ferulic acid***	***–OCH***_***3***_	***—***	***–CH*** = ***CHCOOH***	***✓***	***✓***	***95.6***
***11***	***Sinapic acid***	***2× (–OCH***_***3***_ ***)***	***—***	***–CH*** = ***CHCOOH***	***✓***	***✓***	***76.6***
12	Rutin	–OH and glycosyl^e^	2× (–OH)	Ring C flavonol	✓	—	3.7
13	Ethyl protocatechuate	–OH	—	–COOCH_2_CH_3_	—	—	9.5
14	Myricetin	3× (–OH)	2× (–OH)	Ring C flavonol	✓	—	5.3
15	Resveratrol	—	2× (–OH)	–Vinyl-ring A	✓	—	4.7
16	Quercetin	2× (–OH)	2× (–OH)	Ring C flavonol	✓	—	2.6
17	Kaempferol	–OH	2× (–OH)	Ring C flavonol	✓	—	9.7

a Containing additional electron donating group(s) at the *o*- or *p*-position relative to the *p*-hydroxyl group.

b Conjugation with C=C double bond(s) outside the aromatic ring of acids or ring B of flavonoids.

c Steric of the methoxyl group(s) at the *o*-position(s) hindering oxidation of the *p*-hydroxyl group of the compound.

d Caffeine not containing the *p*-hydroxyl group.

e At the 3 position on the C ring.

A basic concept for the additional functionalities can be obtained from catechol and hydroquinone (*o*- and *p*-dihydroxyphenols, respectively) which were more easily oxidized compared with resorcinol (*m*-dihydroxyphenol).^37^ This is due to the more effective resonance structures donating electron from one to the other –OH of catechol and hydroquinone and providing higher electron density for the oxidation. Larger numbers of electron donating groups in the *o*- or *p*-position(s) also enhance this oxidation. Regardless of the other functional groups, all the compounds with additional –OH group(s) in the effective position(s) relative to the *p*-hydroxyl group showed low recoveries and cannot be the target compounds in this study (see compounds 1, 2, 4, 7, 9 and 12–17 in Table 2).

Although –OCH_3_ is also an electron donating group, the presence of this bulkier functional group (compared with –OH) at *o*-position relative to the *p*-hydroxyl group as mentioned in Table 2 may hinder electron transfer during the oxidation. The presence of the methoxyl group(s) is thus expected to result in the high recoveries of compounds 6, 8, 10 and 11 in Table 2. Such steric effect was also reported for the glycoside group reducing the peak current of rutin compared with that of quercetin,^38^ which could be an explanation herein for the slightly higher recovery of rutin (12) compared with quercetin (16), see Table 2.

In addition, compounds with more extended conjugated structures are expected to show lower oxidation resistance (lower recovery with the EC clean-up). In other words, the analogous compounds with the –CH=CHCOOH group are less stable than that with –COOH due to the extended resonance structure with the vinyl group stabilizing the oxidized form of the former group.^37^ This is illustrated by the lower recovery of sinapic acid (11, 77%) compared with syringic acid (8, 102%). This trend is also expected for flavonoids with pi conjugated system between the B and C rings such as flavonols as well as stilbenes with the conjugated system throughout the A and B rings.^36^ As a result, all the investigated flavonols showed low recoveries from the EC, see compounds 12, 14, 16 and 17 in Table 2. Even without additional electron donating group in the effective positions, resveratrol (15) showed low recoveries due to the presence of extended conjugation system. On the other hand, flavonoids without the conjugated system between the B and C rings such as isoflavanes, flavanone or flavonols are also expected to be stable showing high recovery after the EC. In addition, as discussed above with the steric effects, glycosides of these stable flavonoids are expected with the higher recoveries.

The validated EC approach was further applied for analysis of the survival compounds in three different red wine samples with the results shown in Table 3. Intra-day % RSD and inter-day % RSD of the concentration of the target compounds were within the ranges of 0.24–9.41% and 1.61–15.48%, respectively (Table 3). These low % RSD values for all the wine samples indicate effective sample clean-up performance within 5 s of the EC. Note that the relatively high % RSD of some compounds were caused by the uncertainty in peak integration of the compounds due to coelution with the interference peaks.

**Table 3:** Target compound profiles in different red wine samples analyzed by using the miniaturized EC system at 9.0 V for 5 s and the precisions (*n* = 9). Compounds 3, 18, 6, 19, 8, 20, 10 and 11 were confirmed by the standard injection, and compounds A–H were confirmed by comparison with retention order and UV spectra form the literatures

No.	Compound	*t*_R_ (min)	Calculated concentration (mg L^−1^) or peak area (mAU s) (mean ± SD)			Intra-day precision (% RSD)			Inter-day precision (% RSD)		
			Wine A	Wine B	Wine C	Wine A	Wine B	Wine C	Wine A	Wine B	Wine C
3	*p*-Hydroxybenzoic acid	4.90	0.63 ± 0.03	0.79 ± 0.02	0.75 ± 0.02	2.00	0.75	0.96	4.70	2.41	2.07
18	Catechin	5.65	1.34 ± 0.03	0.96 ± 0.03	2.10 ± 0.06	1.09	1.28	0.24	2.12	3.59	2.95
6	Vanillic acid	6.20	1.23 ± 0.11	2.37 ± 0.08	2.25 ± 0.08	6.02	0.51	9.41	8.94	3.38	10.34
19	*m*-Hydroxybenzoic acid	6.43	15.18 ± 1.40	31.82 ± 2.01	36.84 ± 2.33	7.39	0.41	6.04	9.22	6.33	6.33
8	Syringic acid	7.10	2.13 ± 0.08	1.94 ± 0.12	4.04 ± 0.28	0.41	0.70	0.98	3.80	6.33	7.05
20	(−)-Epicatechin	8.28	1.10 ± 0.02	1.01 ± 0.04	1.30 ± 0.02	1.53	0.91	0.64	1.61	4.05	1.62
10	Ferulic acid	12.63	0.55 ± 0.06	0.54 ± 0.03	0.52 ± 0.01	1.00	2.50	0.61	10.05	6.42	2.86
11	Sinapic acid	13.30	3.93 ± 0.06	NA^a^	1.10 ± 0.10	1.34	NA	7.14	1.66	NA	8.69
A	Indole-3-acetic acid	9.09	39.07 ± 2.01^b^	32.18 ± 2.18^b^	13.98 ± 2.16^b^	0.52	6.54	1.77	5.15	6.79	15.48
B	*p*-Coumaric acid	10.84	72.25 ± 1.44^b^	59.43 ± 1.93^b^	47.84 ± 4.91^b^	0.70	2.06	8.71	1.99	3.25	10.26
C	Unknown	12.27	6.84 ± 0.33^b^	5.62 ± 0.25^b^	1.87 ± 0.17^b^	2.02	1.31	3.34	4.79	4.38	9.15
D	Unknown	15.12	19.23 ± 0.58^b^	15.68 ± 1.11^b^	21.10 ± 1.49^b^	2.97	4.55	5.13	3.03	7.10	7.05
E	Unknown	17.25	NA	NA	3.03 ± 0.27^b^	NA	NA	6.78	NA	NA	8.99
F	Cinnamic acid	18.29	12.74 ± 0.52^b^	9.67 ± 0.69^b^	17.04 ± 0.90^b^	1.30	1.36	2.62	4.09	7.12	5.26

a Not available due to the target compound peak strongly coeluting with the interference peaks.

b Peak area was reported instead of concentration and further used for % RSD calculation.

## Experimental

### Chemicals, standards and materials

Potassium chloride (AR grade), methanol (HPLC grade), acetic acid (glacial, HPLC grade) were obtained from Merck (Germany). Acetonitrile was purchased from J.T. Baker (USA). Ultrapure water obtained from a Milli-Q system (Millipore, Thailand). Stock standard solutions of 1000 mg L^−1^ (chlorogenic acid, 2,5-dihydroxybenzoic acid, vanillic acid, (−)-epicatechin, ethyl gallate, ferulic acid, quercetin, myricetin, caftaric acid, caffeine, *p*-hydroxybenzoic acid, citric acid, caffeic acid, rutin, ethyl protocatechuate, succinic acid, sinapic acid, catechin, *m*-hydroxybenzoic acid, malic acid, syringic acid, resveratrol, kaempferol, gallic acid, protocatechuic acid from Sigma-Aldrich (Germany, USA, Singapore)) were prepared in methanol and/or 50 : 50 methanol : water. Three different red wine samples (named as wine A, B and C) were purchased from local supermarket. All samples were filtered through 0.22 μm nylon membrane filters and then diluted with the electrolyte before use. A sheet of aluminium with a 0.1 cm thickness and stainless steel springs (0.4 and 0.8 cm diameters) were purchased from local supermarket, Thailand. The aluminium sheet was cut into two rectangular electrodes with the dimension of 2.5 cm × 6.0 cm. Pt electrodes (1.0 cm × 1.0 cm × 0.01 cm) were purchased from Aliexpress (product codePt210, Minihua Store, China). Nylon syringe filters (0.22 μm) were purchased from Thermo Fisher Scientific (USA).

### Electrocoagulation (EC)

Sample (20 mL) of standard mixture or wine in 1.5 mol L^−1^ KCl_(aq)_ was transferred into a 50 mL beaker. Two aluminium electrodes were immersed into the sample to reach the beaker bottom. The electrodes were connected with a power supply *via* copper wires, and EC was performed under a constant voltage of 1.0, 1.5, 2.5 and 4.0 V for 5, 10, 15, 30, 60, 120, 180, 240, 300 min at room temperature. The samples before (Control) and after EC were collected and filtered prior to HPLC-UV analysis.

### Miniaturized electrocoagulation system

The solution (1 mL) of standard mixture or wine sample containing KCl 1.5 mol L^−1^ was transferred into a 2 mL vial. Two stainless steel springs with different sizes were used as electrodes. The smaller one was wrapped with parafilm at the two ends and inserted into the larger one. Then, electrodes were immersed into the solution to reach the vial bottom. Each spring end was passed through a rubber cap to avoid contact between the two electrodes. The larger and the smaller electrodes were used as the cathode and anode, respectively, resulting in the higher current compared with the *vice versa* setup. These electrodes were connected with a power supply with applied voltage of 9 V for 5 s. The samples before and after the miniaturized electrocoagulation system were then analyzed with HPLC-UV at 270 nm.

### High performance liquid chromatography-UV detector (HPLC-UV)

Samples were analyzed using an Agilent 1260 Infinity LC system equipped with an ultraviolet detector (UV) (Agilent Technologies). Samples were separated in a reversed-phase C18 (4.6 mm × 100 mm, 2.7 μm particle diameter; Agilent Technologies, USA) column and a flow rate of 1.0 mL min^−1^. 0.1% v/v acetic acid in water and 0.1% v/v acetic acid in acetonitrile were used as mobile phases A and B, respectively. The gradient elution was set as 8, 10, 12, 25, 30, 90 and 100% v/v B at 0, 3.25, 8, 15, 15.8, 25 and 25.4 min, respectively, and the gradient was set back to 8% v/v B for 6.6 min with the overall analysis time of 32 min before the next analysis.^24^ The separation temperature was kept constant at 30 °C. Samples were diluted 10 times in water and injected (10 μL). The wavelengths were scanned from 190 to 400 nm with the selected wavelengths of 270 nm with the detection bandwidth of 4 nm for chromatographic displays.

Agilent Open LAB CDS (ChemStation Edition) and Microsoft Excel were used for peak integration and data visualization. A peak was identified by authentic standard injection and matched with the UV spectrum library. The relative contents of the compounds in samples were calculated according to their peak areas (mAU s).

## Conclusions

This study established EC process with the miniaturized platform of devices for simple and fast sample clean-up approach prior to HPLC analysis. The possible mechanisms mainly involved reduction/enrichment induced aggregation at the cathode supported by coagulation or adsorption of the interference onto the EC floc and sludge. The application was demonstrated here for the selective removal of polymeric pigment species from wine and improved analysis of the electrochemically stable phenolic acids and flavanols. Good performance and reliable results were obtained with the proposed EC condition allowing the 5 s clean-up process. Since the developed device employed solely two stainless steel spring electrodes and a 9 V battery, this is cost effective and adaptable as green sample clean-up process for other applications in the future, especially for removal of polymeric species and selective analysis of compounds with the electrolysis/electrocoagulation resistance in food and biological samples.

## Conflicts of interest

There are no conflicts to declare.

## Supplementary Material

RA-011-D0RA09089A-s001
