# Supplementary material for: Miniaturized electrocoagulation approach for removal of polymeric pigments and selective analysis of non- and mono-hydroxylated phenolic acids in wine with HPLC-UV
Source: RSC Adv. 2021 Feb 2;11(11):5885–93. doi: 10.1039/d0ra09089a (PMC9235540; doi:10.1039/d0ra09089a)
Supplement: RA-011-D0RA09089A-s001 [file RA-011-D0RA09089A-s001.pdf]

Electronic Supplementary Material (ESI) for RSC Advances.
This journal is © The Royal Society of Chemistry 2021

Supplementary data

Miniaturized electrocoagulation approach for removal of polymeric pigments and selective analysis of non- and mono- hydroxylated phenolic acids in wine with HPLC-UV

Kanokporn Chindaphan[1], Isaya Thaveesangsakulthai[1], Suchapa Naranaruemol[1], Thumnoon Nhujak[1,2], Janjira Panchompoo[1,3], Orawon Chailapakul[1,3] and Chadin Kulsing[1,2*]

[1] Department of Chemistry, Faculty of Science, Chulalongkorn University, Bangkok 10330, Thailand

[2] Chromatographic Separation and Flavor Chemistry Research Unit and Center of Molecular Sensory Science, Department of Chemistry, Faculty of Science, Chulalongkorn University, Bangkok 10330, Thailand

[3] Electrochemistry and Optical Spectroscopy Center of Excellence (EOSCE), Department of Chemistry, Faculty of Science, Chulalongkorn University, Bangkok 10330, Thailand

***Corresponding Author.**

Chromatographic Separation and Flavor Chemistry Research Unit and Center of Molecular Sensory Science, Department of Chemistry, Faculty of Science, Chulalongkorn University, Bangkok 10330, Thailand

Tel.: +6802971178; Fax: +6622187598

Email address: ckulsing@gmail.com (C. Kulsing)

Experimental

S1. Electrolysis

Wine sample (5 mL) was filtrated using nylon syringe filter and transferred into a 10 mL beaker. The electrolysis cell consisted of two Pt electrodes connected with a DC power supply via copper wires to complete a circuit and immersed into the wine sample. The electrode positions were fixed by a foam cap containing the channels with dimension of the cross sectional electrode holder. Electrolysis was performed under a constant voltage at room temperature (28±1 °C). The samples before and after the electrolysis for 30, 60, 120, 180, 240, 300 min were collected and filtered prior to HPLC-UV analysis.

S2. Differential pulse voltammetry (DPV)

The wine samples were prepared by addition of 0.50 mL of red wine A, 0.33 mL of 3 mol L^{-1} KCl and 9.17 mL of Milli-Q water, respectively in a 10 mL beaker. Electrochemical experiments were performed using a potentiostat (PGSTAT10) with Nova software, version 2.1.2. DPV conditions with pulse amplitude of 100 mV, pulse width of 125 ms and scan rate of 4.96 mV s^{-1} were performed with the potential starting from 0.1 to -1.2 V for reduction. Differential pulse voltammograms were recorded at room temperature using a three-electrode system in a 10 mL beaker with a foam cap. The reference electrode was Ag/AgCl (in saturated KCl). The working electrode (at which the desired reduction occurred) was the larger stainless steel spring while the smaller stainless steel spring acted as the counter electrode (where the associated oxidation would take place).

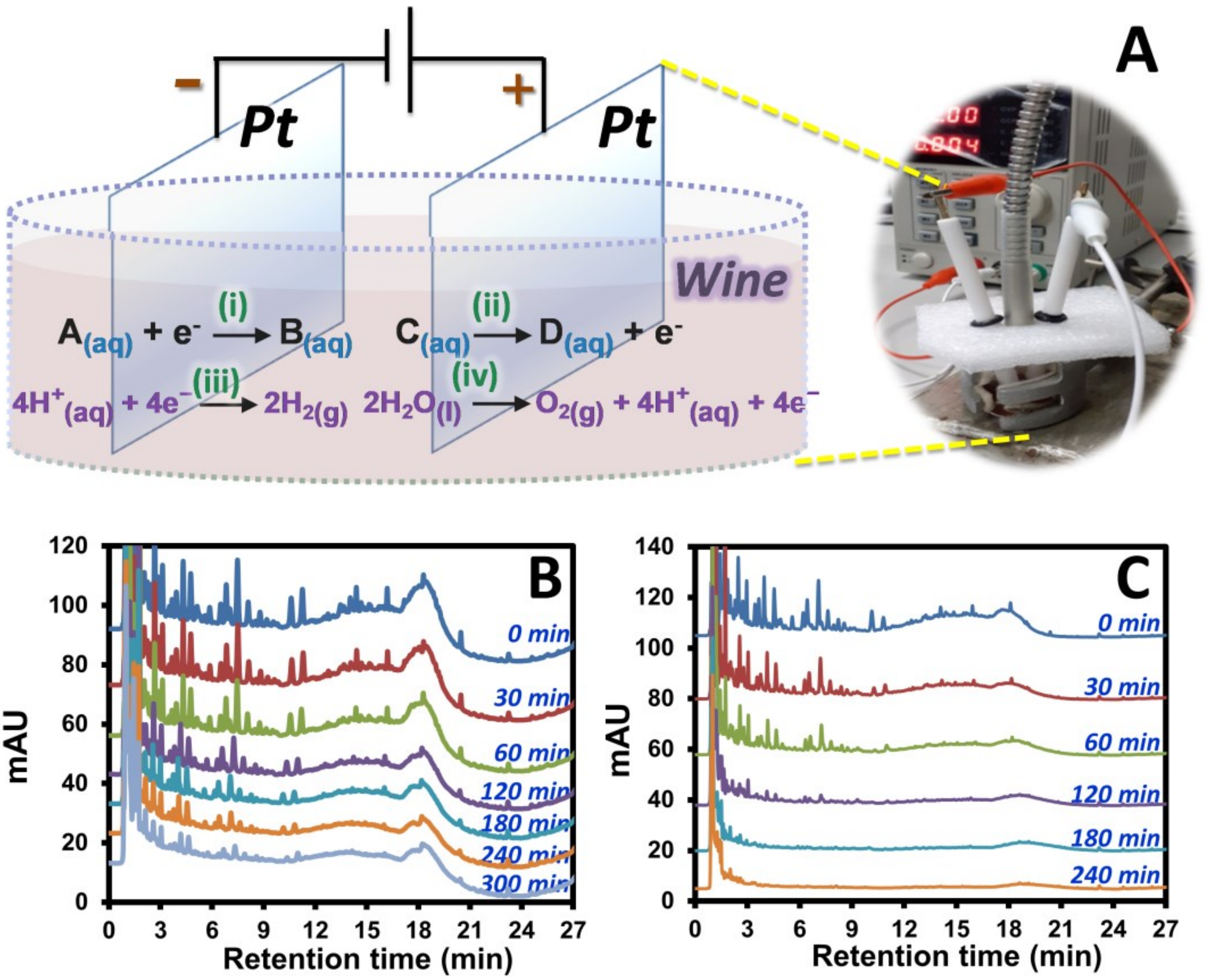

Fig. S1 Overall bulk electrolysis studies: (A) experimental setup and chromatograms at 270 nm of (B) wine A sample collected at different electrolysis time at 9.0 V and (C) wine A sample containing 2.6 mol L^{-1} of NaCl(aq) collected at different electrolysis time at 4.0 V. The related mechanisms in A are (i) reduction and (ii) oxidation of wine compounds, (iii) proton reduction and (iv) water oxidation. A and C represent compounds undergoing electrochemical reaction into compounds B and D, respectively.

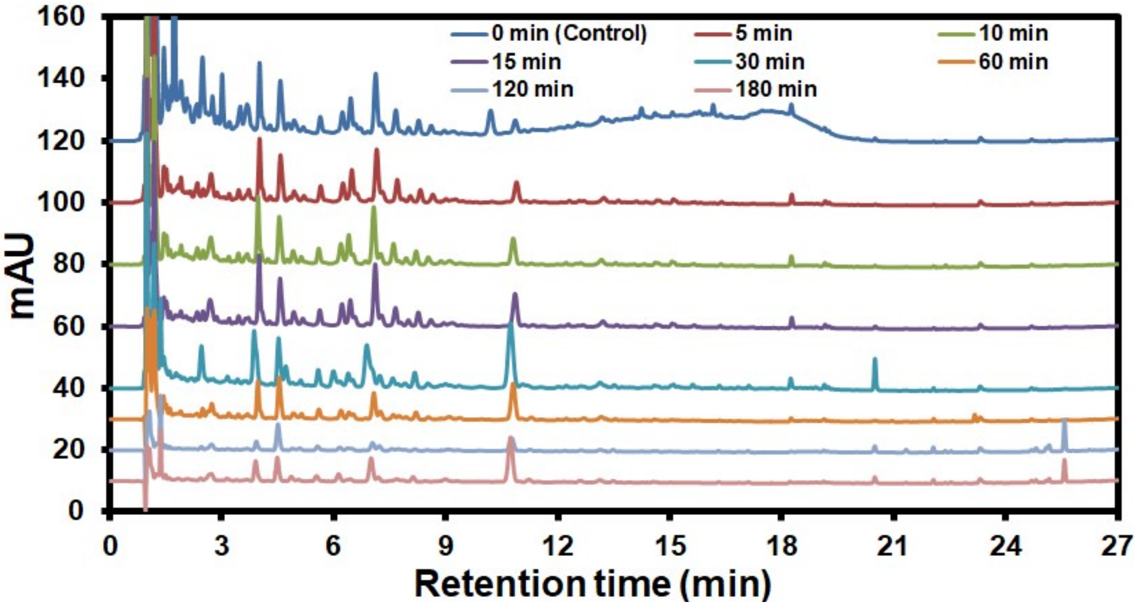

Fig. S2. Chromatograms at 270 nm of wine A sample containing 1.5 mol L^{-1} of $KCl_{(aq)}$ collected at different time of bulk electrocoagulation at 2.5 V.

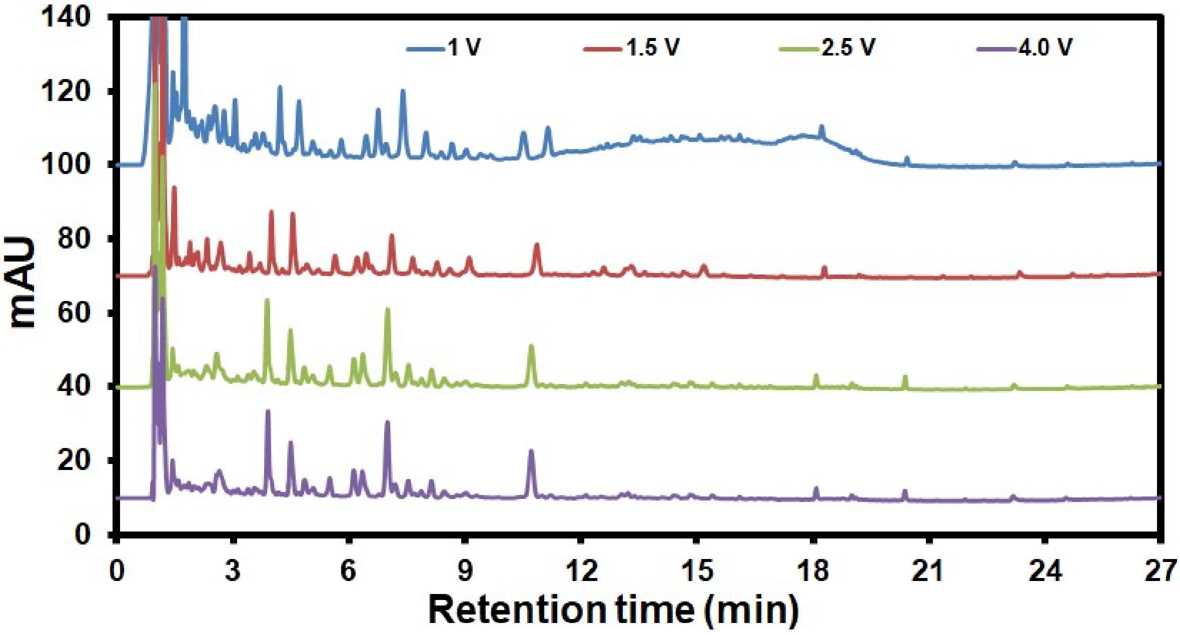

Fig. S3. Chromatograms at 270 nm of wine A sample containing 1.5 mol L^{-1} of $KCl_{(aq)}$ collected at 30 min of bulk electrocoagulation using different voltage.

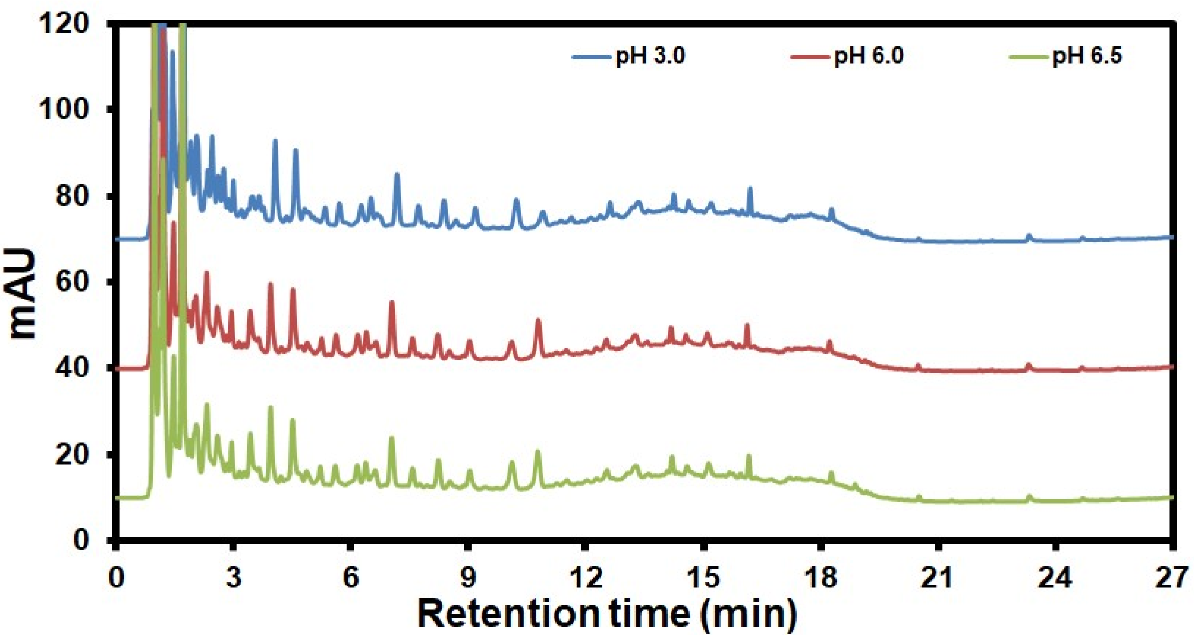

Fig. S4. Chromatograms at 270 nm of wine A sample containing 1.5 mol L^{-1} of $KCl_{(aq)}$ adjusted to different pH without EC.

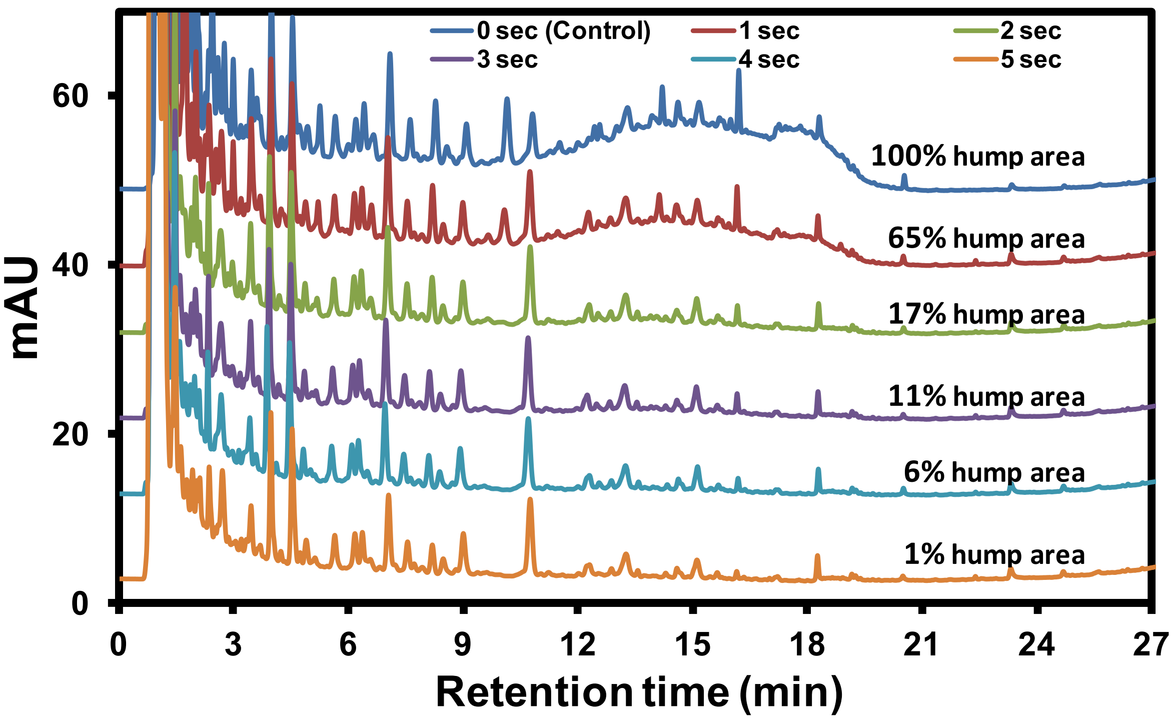

Fig. S5. Chromatograms at 270 nm of wine A sample containing 1.5 mol L^{-1} of $KCl_{(aq)}$ collected at different time of miniaturized electrocoagulation at 9.0 V with the percentage of interference (hump) area relative to that of the control.

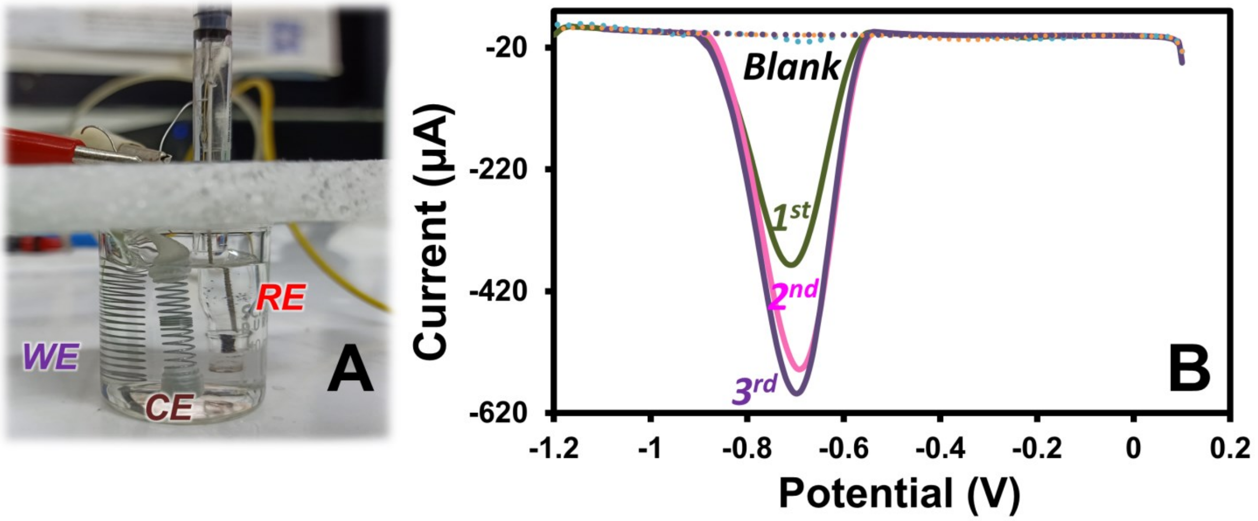

Fig. S6 Overall DPV analysis of wine A containing 0.1 mol L^{-1} of KCl: (A) experimental setup, and (B) Differential pulse voltammograms (only recorded for the first scan) of wine A sample (20-fold dilution in 0.1 mol L^{-1} of KCl(aq)) and blank solution (0.6% EtOH in 0.1 mol L^{-1} of KCl) (scanned potential negatively from 0.1 to -1.2 V), at a scan rate of 4.96 mV s^{-1}. The 1st, 2nd and 3rd measurements indicate the replicate analyses.

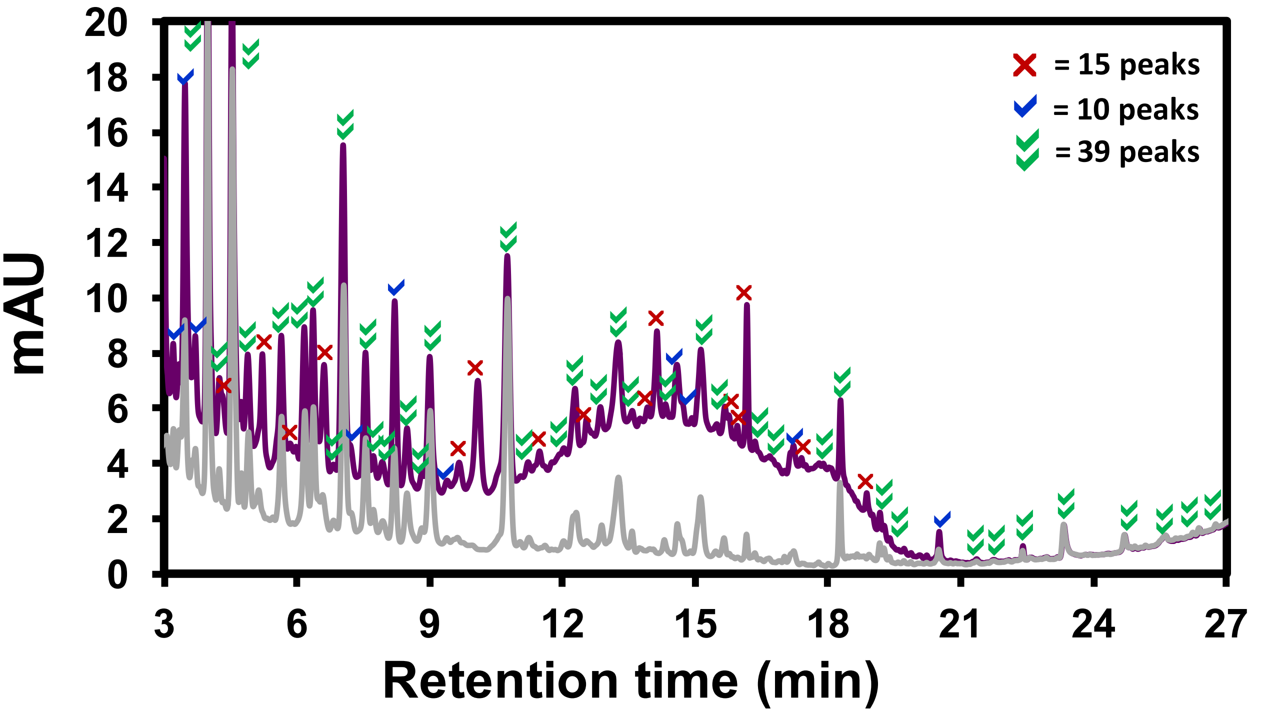

Fig. S7 Chromatograms at 270 nm of wine A sample containing 1.5 mol L^{-1} of $KCl_{(aq)}$ before (above) and after (below) the EC cleanup. ×, ✓ and ✓✓ indicate peaks detected with low, medium and high areas after the EC cleanup compared with the corresponding peaks before the cleanup.
